# Supplementary material for: Mid-Cretaceous marine Os isotope evidence for heterogeneous cause of oceanic anoxic events
Source: Nat Commun. 2022 Jan 11;13:239. doi: 10.1038/s41467-021-27817-0 (PMC8752794; doi:10.1038/s41467-021-27817-0)
Supplement: Supplementary file 1 — Supplementary Information [file 41467_2021_27817_MOESM1_ESM.pdf]

Supplementary information for "Mid-Cretaceous marine Os isotope stratigraphy for the evolutional history of hydrothermal activity"

Hironao Matsumoto[1*], R. Coccioni[2], F. Frontalini[3], Kotaro Shirai[1], Luigi Jovane[4], Ricardo Trindade[5], Jairo F. Savian[6], J. Kuroda[1]

[1]*Atmosphere and Ocean Research Institute, The University of Tokyo, 5-1-5 Kashiwanoha, Kashiwa 277-8564, Japan*
[2]*University of Urbino, Carlo Bo, 61029 Urbino, Italy*
[3]*DiSPeA, University of Urbino Carlo Bo, Campus Scientifico Enrico Mattei, Località Crocicchia, 61029 Urbino, Italy*
[4]*Instituto Oceanográfico, Universidade de São Paulo, Praça do Oceanográfico, 191 - São Paulo, SP 05508-120, Brazil*
[5]*Instituto de Astronomia, Geofísica e Ciências Atmosféricas, Universidade de São Paulo, Rua do Matão, 1226 - São Paulo, SP 05508-090, Brazil*
[6]*Departamento de Geologia, Instituto de Geociências, Universidade Federal do Rio Grande do Sul, Avenida Bento Gonçalves, 9500 – Porto Alegre, RS 91501-970, Brazil*

Information of corresponding author:
Emai: matsumoto@aori.u-toyko.ac.jp
Address: *Atmosphere and Ocean Research Institute, Japan, The University of Tokyo, 5-1-5 Kashiwanoha, Kashiwa 277-8564, Japan*

Inventory of Supplementary Information

- Supplementary Table 1: Carbon and oxygen isotopic ratios of carbonate of PLG core
- Supplementary Table 2: Carbon and oxygen isotopic ratios of carbonate of Bottaccione section
- Supplementary Table 3: Carbon and oxygen isotopic ratios of carbonate of ODP Site 763B
- Supplementary Table 4: Re- and Os- abundance and Os isotopic ratios of carbonate of PLG core samples
- Supplementary Table 5: Re- and Os- abundance and Os isotopic ratios of carbonate of Bottaccione section samples
- Supplementary Table 6: Re- and Os- abundance and Os isotopic ratios of carbonate of ODP Site 763B samples

Supplementary Table 1: Carbon and oxygen isotopic ratio of carbonate of PLG core

Sample Name	$\delta^{13}C_{carb}$	1SD	$\delta^{18}O_{carb}$	1SD	Depth
	(‰VPDB)		(‰VPDB)		(mbsf)
PLG-C 0.25	2.55	0.05	-2.22	0.05	92.86
PLG-C 1.43	2.41	0.07	-1.99	0.08	91.68
PLG-C 1.85	2.25	0.05	-2.84	0.05	91.26
PLG-C 2.26	2.32	0.07	-2.16	0.08	90.85
PLG-C 2.39	2.32	0.05	-2.70	0.05	90.72
PLG-C 3.54	2.23	0.07	-2.14	0.08	89.57
PLG-C 4.50	2.16	0.05	-2.35	0.05	88.61
PLG-C 5.37	2.19	0.07	-2.12	0.08	87.74
PLG-C 6.21	2.22	0.07	-1.77	0.08	86.90
PLG-C 7.10	2.14	0.05	-1.75	0.05	86.01
PLG-C 8.02	2.23	0.05	-2.46	0.05	85.09
PLG-C 8.66	2.28	0.05	-2.15	0.05	84.45
PLG-C 9.16	2.30	0.07	-1.84	0.08	83.95
PLG-C 9.56	2.38	0.07	-1.82	0.08	83.55
PLG-C 10.31	2.09	0.05	-1.95	0.05	82.80
PLG-C 10.75	2.24	0.07	-1.90	0.08	82.36
PLG-C 11.81	2.30	0.05	-2.18	0.05	81.30
PLG-C 12.60	2.39	0.07	-1.44	0.08	80.51
PLG-C 13.76	2.58	0.05	-2.62	0.05	79.35
PLG-C 14.44	2.52	0.07	-2.64	0.08	78.67
PLG-C 14.58	2.54	0.05	-2.20	0.05	78.53
PLG-C 15.36	2.76	0.07	-2.05	0.08	77.75
PLG-C 16.32	2.58	0.05	-2.35	0.05	76.79
PLG-C 17.44	2.51	0.07	-1.50	0.08	75.67
PLG-C 18.22	2.37	0.05	-2.12	0.05	74.89
PLG-C 19.20	2.63	0.07	-2.17	0.08	73.91
PLG-C 19.42	2.29	0.05	-1.94	0.05	73.69
PLG-C 20.35	2.43	0.07	-1.82	0.08	72.76
PLG-C 21.20	2.64	0.05	-2.51	0.05	71.91
PLG-C 22.12	2.72	0.07	-2.17	0.08	70.99
PLG-C 23.13	2.54	0.05	-2.29	0.05	69.98
PLG-C 25.00	2.40	0.11	-2.00	0.08	68.11
PLG-C 26.18	2.56	0.05	-1.94	0.05	66.93
PLG-C 27.25	2.58	0.11	-1.69	0.08	65.86
PLG-C 28.27	2.83	0.05	-2.08	0.05	64.84
PLG-C 29.26	2.84	0.11	-2.10	0.08	63.85
PLG-C 30.30	2.63	0.05	-1.97	0.05	62.81
PLG-C 31.33	2.68	0.11	-2.04	0.08	61.78
PLG-C 32.08	2.69	0.05	-1.83	0.05	61.03
PLG-C 33.09	2.77	0.11	-2.23	0.08	60.02
PLG-C 34.09-34.10	2.77	0.05	-2.02	0.05	59.02
PLG-C 35.20	2.93	0.11	-2.26	0.08	57.91
PLG-C 36.00	2.65	0.05	-1.89	0.05	57.11
PLG-C 36.88	2.53	0.11	-2.13	0.08	56.23
PLG-C 38.02	2.67	0.05	-2.15	0.05	55.09
PLG-C 39.09	2.60	0.11	-2.37	0.08	54.02
PLG-C 39.93	2.92	0.05	-2.43	0.05	53.18
PLG-C 40.92	2.62	0.11	-2.35	0.08	52.19
PLG-C 42.01-42.05	2.90	0.05	-2.38	0.05	51.10
PLG-C 42.48-42.49	2.68	0.11	-2.41	0.08	50.63
PLG-C 43.70	2.86	0.05	-2.26	0.05	49.41
PLG-C 45.00-45.03	2.86	0.05	-2.23	0.05	48.11
PLG-C 45.98	2.59	0.11	-2.21	0.08	47.13
PLG-C 47.03	2.13	0.11	-2.41	0.08	46.08
PLG-C 48.01	3.00	0.05	-2.23	0.05	45.10
PLG-C 48.99	2.92	0.11	-2.08	0.08	44.12
PLG-C 50.00	2.99	0.05	-1.97	0.05	43.11
PLG-C 50.98-50.99	2.97	0.11	-2.19	0.08	42.13

Supplementary Table 2: Carbon and oxygen isotopic ratio of carbonate of Bottaccione section

Sample Name	δ^{13}Ccarb (‰VPDB)	1SD	δ^{18}Ocarb (‰VPDB)	1SD	Depth (mbsf)
BTT 561	2.55	0.11	-3.18	0.08	143.92
BTT 548	2.43	0.05	-3.56	0.05	142.79
BTT 521	2.44	0.11	-3.34	0.08	139.76
BTT 484	2.46	0.05	-3.16	0.05	134.13
BTT 463	2.30	0.11	-3.02	0.08	130.85
BTT 434	2.59	0.05	-3.22	0.05	127.63
BTT 404	2.19	0.05	-3.47	0.05	123.86
BTT 372	2.06	0.11	-3.04	0.08	119.91
BTT 318	2.03	0.11	-2.81	0.08	113.99
BTT 268	2.11	0.05	-2.86	0.05	108.46
BTT 225	2.03	0.11	-3.31	0.08	104.53
BTT160	2.67	0.05	-3.04	0.05	98.66
BTT131	2.37	0.11	-3.18	0.08	95.39
BTT 107	2.59	0.05	-2.90	0.05	93.35

38

Supplementary Table 3: Carbon and oxygen isotopic ratio of carbonate of ODP Site 763B

Sample Name	$\delta^{13}C_{carb}$	1SD	$\delta^{18}O_{carb}$	1SD	Depth
	(‰VPDB)		(‰VPDB)		(mbsf)
763B_36-7_14-17	3.05	0.07	-0.50	0.08	521.79
763B_36-6_100-103	3.04	0.05	-1.12	0.05	521.15
763B_36-5_117-120	3.16	0.07	-1.43	0.08	519.82
763B_36-4_81-84	3.77	0.05	-1.59	0.05	518.31
763B_36-3_30-33	3.91	0.07	-1.34	0.08	516.3
763B_36-2_93-96	3.68	0.05	-1.82	0.05	515.43
763B_36-1_94-97	3.90	0.07	-1.53	0.08	513.94
763B_35-CC_41-43	4.09	0.05	-1.80	0.05	512.91
763B_35-6_115-119	3.42	0.07	-1.95	0.08	512.15
763B_35-5_129-133	3.23	0.05	-2.22	0.05	510.79
763B_35-4_121-126	3.72	0.07	-1.36	0.08	509.21
763B_35-3_124-128	3.71	0.05	-1.91	0.05	507.74
763B_35-2_99-102	2.99	0.07	-1.11	0.08	505.99
763B_35-1_81-84	3.54	0.05	-1.90	0.05	504.31
763B_34-7_9-14	3.12	0.07	-2.57	0.08	503.09
763B_34-6_14-18	2.77	0.11	-2.39	0.08	501.64
763B_34-5_17-23	3.52	0.07	-1.82	0.08	500.17
763B_34-4_94-98	3.27	0.11	-1.82	0.08	499.44
763B_34-3_42-46	3.51	0.07	-1.51	0.08	497.42
763B_34-2_50-53	3.03	0.07	-1.98	0.07	496
763B_34-1_27-29	3.31	0.11	-1.79	0.08	494.27
763B_33-7_24-27	3.43	0.07	-1.91	0.08	493.39
763B_33-6_30-32	3.80	0.11	-1.49	0.08	491.95
763B_33-5_40-43	3.08	0.07	-1.83	0.08	490.9
763B_33-4_2-5	3.62	0.11	-2.09	0.08	489.02
763B_33-3_7-10	2.67	0.07	-2.06	0.08	487.57
763B_33-2_56-59	3.12	0.11	-2.36	0.08	486.56
763B_32-CC_15-19	3.28	0.11	-1.80	0.08	484.6
763B_33-1_5-8	3.26	0.07	-2.35	0.08	484.55
763B_32-7_14-17	3.27	0.07	-2.00	0.08	484.14
763B_32-6_45-51	1.24	0.11	-2.17	0.08	482.95
763B_32-5_29-33	2.22	0.07	-2.32	0.07	481.29
763B_32-4_49-52	1.72	0.07	-2.10	0.08	479.99
763B_32-3_43-48	2.64	0.11	-2.40	0.08	478.43
763B_32-2_30-32	2.87	0.07	-2.58	0.08	476.8
763B_32-1_60-63	2.20	0.11	-2.53	0.08	475.6
763B_31-CC_28-33	3.11	0.07	-2.47	0.08	475.155
763B_31-7_1-6	2.05	0.11	-2.14	0.08	474.51
763B_31-6_25-29	3.45	0.07	-2.16	0.08	473.25
763B_31-5_26-28	2.93	0.11	-1.87	0.08	471.76
763B_31-4_63-65	1.43	0.07	-1.96	0.08	470.63
763B_31-3_48-52	1.88	0.11	-1.96	0.08	468.98
763B_31-2_36-39	-1.33	0.07	-1.85	0.08	467.36
763B_31-1_9-12	2.40	0.11	-2.00	0.08	465.59
763B_30-CC_39-41	2.46	0.07	-1.83	0.08	465.44
763B_30-7_32-35	1.93	0.11	-2.36	0.08	464.97
763B_30-6_33-37	2.14	0.07	-1.72	0.08	463.48
763B_30-5_20-24	1.90	0.07	-1.82	0.07	461.85
763B_30-4_78-83	2.27	0.11	-2.10	0.08	461.28
763B_30-3_41-44	-0.40	0.07	-1.87	0.08	459.41
763B_30-2_64-68	1.79	0.07	-1.96	0.07	458.14
763B_29-CC_72-75	2.46	0.07	-2.03	0.08	456.67
763B_30-1_45-47	1.81	0.11	-2.35	0.08	456.45
763B_29-7_0-5	2.67	0.11	-2.31	0.08	455.5
763B_29-6_43-48	1.85	0.07	-2.11	0.08	454.43
763B_29-5_85-88	2.25	0.07	-2.12	0.07	453.35
763B_29-4_33-36	2.06	0.11	-2.13	0.08	451.33
763B_29-3_52-56	2.30	0.07	-2.14	0.08	450.02
763B_29-2_17-21	2.90	0.07	-2.37	0.07	448.17
763B_29-1_51-54	2.73	0.11	-2.62	0.08	447.01
763B_28-CC_16-21	2.73	0.07	-2.75	0.08	446.61
763B_28-7_16-19	2.27	0.11	-2.54	0.08	446.16
763B_28-6_55-59	2.33	0.07	-2.37	0.08	445.05
763B_28-5_54-57	2.23	0.07	-2.38	0.07	443.54
763B_28-4_40-44	2.49	0.11	-2.37	0.08	441.9
763B_28-3_30-34	3.04	0.07	-2.53	0.08	440.3
763B_28-2_20-23	2.02	0.07	-2.91	0.07	438.7
763B_28-1_7-11	1.87	0.11	-2.95	0.08	437.07
763B_27-CC_21-24	1.64	0.07	-2.50	0.08	436.685
763B_27-7_27-32	1.91	0.11	-2.48	0.08	436.42
763B_27-6_64-68	0.45	0.07	-2.79	0.08	435.29
763B_27-5_115-116	1.94	0.07	-2.96	0.07	434.3
763B_27-4_52-55	1.64	0.11	-2.51	0.08	432.52
763B_27-3_29-32	1.56	0.07	-3.32	0.08	430.79
763B_27-2_25-28	1.52	0.07	-2.59	0.07	429.25
763B_27-1_64-66	2.30	0.11	-2.64	0.08	428.14
763B_26-CC_29-32	2.08	0.07	-2.63	0.08	424.74
763B_26-5_6-9	1.39	0.07	-2.46	0.07	424
763B_26-4_60-63	2.57	0.11	-2.63	0.08	423.1
763B_26-3_44-47	0.67	0.07	-2.42	0.08	421.44
763B_26-2_48-51	1.74	0.07	-2.27	0.07	419.98
763B_25-CC_52-54	2.28	0.07	-2.55	0.08	418.345
763B_26-1_44-47	2.88	0.11	-2.29	0.08	418.265
763B_25-7_14-17	2.29	0.11	-2.43	0.08	417.64
763B_25-6_33-36	2.83	0.07	-2.18	0.08	416.33
763B_25-5_23-26	2.52	0.07	-2.54	0.07	414.73
763B_25-4_31-35	2.90	0.11	-2.90	0.08	413.31
763B_25-3_16-19	3.31	0.07	-2.36	0.08	411.66
763B_25-2_35-38	2.47	0.07	-2.43	0.07	410.35
763B_25-1_92-94	2.24	0.11	-2.34	0.08	409.42
763B_24-CC_10-12	3.57	0.07	-2.26	0.08	408.2
763B_24-7_27-30	2.50	0.11	-2.34	0.08	407.92
763B_24-6_36-38	1.99	0.07	-2.85	0.08	406.51
763B_24-5_25-28	3.00	0.07	-2.56	0.07	405.25
763B_24-4_57-60	2.20	0.11	-2.88	0.08	404.07
763B_24-3_60-63	1.86	0.07	-2.33	0.08	402.6
763B_24-2_39-40	1.54	0.07	-2.69	0.07	400.89
763B_24-1_56-58	1.24	0.11	-2.48	0.08	399.56
763B_23-CC_15-18	0.99	0.07	-2.43	0.08	396.725
763B_23-5_10-14	-0.79	0.11	-2.11	0.08	395.6
763B_23-4_76-80	1.63	0.07	-2.24	0.07	394.76
763B_23-3_43-46	0.85	0.07	-2.25	0.08	392.93
763B_23-2_6-9	2.61	0.07	-2.40	0.07	391.06
763B_23-1_24-27	-0.45	0.11	-1.87	0.08	389.74
763B_22-CC_28-30	-1.79	0.07	-1.80	0.07	381.98
763B_22-2_3-7	-2.74	0.07	-1.80	0.08	381.53
763B_22-1_42-43	-1.66	0.07	-1.94	0.07	380.42
763B_21-CC_16-19	1.02	0.07	-2.19	0.07	372.96
763B_21-2_66-69	-0.40	0.11	-1.54	0.08	372.66
763B_21-1_42-45	-0.35	0.07	-1.59	0.07	370.92

Supplementary Table 4: Re and Os abundance and Os isotopic ratios of Poggio le Guaine Core samples

Sample Name	Lithology	Os (pg g^{-1})	1sd	$^{187}Os/^{188}Os$	1sd	Re conc (pg g^{-1})	1sd	$^{187}Re/^{188}Os$	1sd	$^{187}Os/^{188}Os_{initial}$	1sd	Depth (m)	Age (Ma)
PGC2.26	Marlstone	25.13	0.17	0.716	0.010	52	3	10.7	0.6	0.698	0.010	90.23	101.1
PGC-3.54	Marlstone	20.7	0.4	0.686	0.020	1	8	0.4	1.9	0.69	0.02	88.95	101.4
PGC-5.37	Marlstone	15.09	0.14	0.701	0.011	16	3	5.4	1.0	0.692	0.011	87.12	101.7
PGC-8.02	Marlstone	35.65	0.18	0.677	0.009	15	3	2.1	0.4	0.674	0.009	84.47	102.2
PLGC-9.16	Marlstone	22.38	0.13	0.645	0.010	10	2	2.2	0.6	0.642	0.010	83.33	102.4
PGC-12.60	Marlstone	14.26	0.17	0.678	0.015	10	3	3.5	1.0	0.672	0.016	79.89	103.1
PGC-15.36	Marlstone	19.4	0.3	0.701	0.016	150	7	40.0	1.9	0.632	0.017	77.13	103.6
PGC-17.44	Marlstone	22.38	0.16	0.626	0.011	190	5	43.5	1.2	0.550	0.011	75.05	104.0
PLG-C 18.22	Marlstone	21.87	0.16	0.458	0.009	162	4	37.2	0.9	0.394	0.009	74.27	104.2
PGC-19.10	Marlstone	542	4	0.593	0.005	3449	19	32.5	0.3	0.536	0.005	73.39	104.3
PGC-22.12	Marlstone	40.6	0.3	0.577	0.008	60	12	7.6	1.5	0.563	0.009	70.37	104.9
PGC-24.68	Marlstone	138.0	0.9	0.798	0.009	3081	20	116.9	1.1	0.592	0.009	67.81	105.4
PGC-27.25	Marlstone	21.2	0.2	0.623	0.013	19	5	4.6	1.3	0.615	0.013	65.24	105.9
PGC-29.26	Marlstone	29.28	0.18	0.483	0.006	17	3	2.8	0.5	0.478	0.006	63.23	106.3
PGC-30.30	Marlstone	23.95	0.16	0.492	0.010	51	3	10.8	0.6	0.473	0.010	62.19	106.5
PGC-33.09	Marlstone	47.9	0.3	0.445	0.006	215	7	22.5	0.8	0.405	0.006	59.4	107.0
PGC-35.20	Marlstone	45.6	0.4	0.565	0.013	6	7	0.7	0.8	0.563	0.013	57.29	107.4
PGC-36.88	Marlstone	41.2	0.3	0.664	0.011	271	9	33.8	1.1	0.603	0.011	55.61	107.7
PGC-39.09	Shale	36.4	0.3	0.624	0.008	217	6	30.5	0.8	0.569	0.008	53.4	108.1
PLG-C40.92	Marlstone	34.1	0.2	0.613	0.008	282	7	42.4	1.1	0.537	0.008	51.57	108.5
PGC-43.70	Marlstone	48.8	0.3	0.618	0.008	90	4	9.4	0.4	0.601	0.008	48.79	109.0
PGC-45.98	Marlstone	29.4	0.3	0.682	0.013	335	16	59	3	0.574	0.014	46.51	109.5
PGC-48.01	Marlstone	34.3	0.2	0.629	0.009	79	3	11.8	0.5	0.608	0.009	44.49	109.8
PGC-50.00	Marlstone	16.44	0.13	0.651	0.013	25	3	7.8	1.0	0.637	0.013	42.49	110.2

Supplementary Table 5: Re and Os abundance and Os isotopic ratios of Bottaccione samples

Sample Name	Lithology	Os (pg g-1)	1sd	$^{187}Os/^{188}Os$	1sd	Re (pg g-1)	1sd	$^{187}Re/^{188}Os$	1sd	$^{187}Os/^{188}Os_i$	1sd	Depth (m)	Age (Ma)
BT107	Limestone	12.30	0.10	0.713	0.013	5.5	1.8	2.3	0.8	0.709	0.013	93.35	100.55
BTT120	Limestone	13.11	0.09	0.682	0.008	4	2	1.4	0.9	0.679	0.008	94.38	100.42
BTT131	Limestone	15.07	0.13	0.721	0.014	3	3	1.0	1.1	0.719	0.014	95.39	100.29
BTT151	Limestone	21.25	0.18	0.720	0.012	8	3	1.9	0.6	0.717	0.012	97.54	100.02
BT160	Limestone	12.73	0.12	0.700	0.015	10	3	4.2	1.3	0.693	0.015	98.66	99.88
BT225	Limestone	11.83	0.13	0.734	0.016	6.5	1.5	2.8	0.7	0.729	0.016	104.53	99.14
BT268	Limestone	9.98	0.11	0.748	0.014	4.1	1.8	2.1	0.9	0.744	0.014	108.46	98.64
BT318	Limestone	10.64	0.12	0.752	0.015	5.4	1.7	2.6	0.8	0.747	0.015	113.99	97.94
BT372	Limestone	10.77	0.12	0.714	0.013	1	2	0.6	1.1	0.713	0.013	119.91	97.20
BT404	Limestone	17.29	0.16	0.720	0.009	2	2	0.6	0.7	0.719	0.009	123.86	96.70
BT434	Limestone	10.42	0.16	0.703	0.017	2	3	1.1	1.4	0.701	0.017	127.63	96.22
BTT450*	Limestone	74.5	0.5	1.152	0.011	144	3	10.6	0.2	1.135	0.011	129.51	95.99
BT463	Limestone	14.60	0.17	0.627	0.013	4	2	1.4	0.8	0.625	0.013	130.85	95.82
BTT474	Limestone	12.15	0.12	0.763	0.014	2	3	0.9	1.2	0.761	0.014	132.76	95.58
BT484	Limestone	13.76	0.14	0.781	0.013	10	2	3.9	0.8	0.775	0.013	134.13	95.40
BTT496	Limestone	13.31	0.14	0.670	0.012	8	3	3.2	1.2	0.665	0.013	136.14	95.15
BTT508	Limestone	13.42	0.13	0.652	0.013	2	3	0.7	1.1	0.651	0.013	137.99	94.92
BT521	Limestone	16.56	0.13	0.672	0.011	6	3	2.0	0.8	0.669	0.011	139.76	94.69
BTT542	Limestone	15.63	0.15	0.699	0.010	2	2	0.6	0.8	0.698	0.010	141.93	94.42
BTT552	Limestone	15.57	0.10	0.549	0.007	3	2	0.9	0.8	0.547	0.007	143.13	94.27
BT561B	Limestone	29.06	0.13	0.375	0.005	19.4	1.9	3.3	0.3	0.370	0.005	143.92	94.17

* excluded from the discussion

Supplementary Table 6: Re and Os abundance and Os isotopic ratios of ODP Site 763B

Sample Name	Lithology	Os	1sd	$^{187}Os/^{188}Os$	1sd	Re	1sd	$^{187}Re/^{188}Os$	1sd	$^{187}Os/^{188}Os_i$	1sd	Depth	Age
		(pg g-1)				(pg g-1)						(m)	(Ma)
763B_36-3_138-141	Nannofossil claystone	38.0	0.3	0.704	0.008	85	2	11.6	0.2	0.682	0.008	517.38	111.25
763B_35-4_121-126	Nannofossil claystone	45.8	0.2	0.605	0.010	106	3	11.8	0.3	0.583	0.010	509.21	110.07
763B_35-2_99-102	Nannofossil claystone	47.1	0.5	0.633	0.010	37.9	1.1	4.1	0.13	0.625	0.010	505.99	109.60
763B_34-6_111-115	Calcareous claystone	40.1	0.2	0.640	0.007	113	3	14.5	0.4	0.614	0.007	502.61	109.10
763_34-2_50-53	Calcareous claystone	45.3	0.3	0.609	0.008	250	6	28.2	0.7	0.558	0.008	496	108.14
763B_33-6_115-118	Calcareous claystone	53.2	0.3	0.585	0.008	80	3	7.7	0.3	0.571	0.008	492.8	107.68
763B_33-3_7-10	Calcareous claystone	54.4	0.2	0.510	0.007	242	7	22.5	0.6	0.470	0.007	487.57	106.92
763_32-5_29-33	Calcareous claystone	44.8	0.3	0.609	0.008	119	4	13.6	0.4	0.585	0.008	481.29	106.00
763B_32-1_125-129	Calcareous claystone	33.94	0.16	0.682	0.007	93	2	14.2	0.3	0.658	0.007	476.25	105.27
763B_31-6_25-29	Calcareous claystone	51.6	0.2	0.669	0.005	251	3	25.1	0.4	0.625	0.005	473.25	104.83
763B_31-3_48-52	Calcareous claystone	40.49	0.17	0.512	0.007	156	4	19.5	0.5	0.478	0.007	468.98	104.21
763B_31-1_9-12	Calcareous claystone	25.5	0.2	0.711	0.011	348	4	70.7	1.1	0.589	0.011	465.59	103.72
763B_30-5_115-118	Nannofossil claystone	22.69	0.14	0.712	0.010	91	2	20.8	0.5	0.676	0.010	462.8	103.31
763_30-3_41-44	Nannofossil claystone	18.3	0.4	0.70	0.03	138	5	39.1	1.5	0.64	0.03	459.41	102.82
763B_29-6_110-113	Calcareous claystone	34.25	0.17	0.699	0.008	179	3	27.0	0.5	0.653	0.008	455.1	102.19
763B_29-3_104-109	Calcareous claystone	21.7	0.2	0.738	0.011	90	4	21.6	0.9	0.702	0.012	450.54	101.53
763B_29-1_51-54	Calcareous claystone	22.98	0.11	0.735	0.010	152	2	34.3	0.6	0.677	0.010	447.01	101.01
763B_28-4_40-44	Calcareous claystone	24.35	0.11	0.709	0.007	133	3	28.2	0.6	0.662	0.007	441.9	100.33
763_28-1_7-11	Calcareous claystone	20.3	0.2	0.71	0.02	152	5	38.9	1.5	0.65	0.02	437.07	99.81
763B_27-4_108-111	Nannofossil claystone	22.42	0.14	0.748	0.017	143	3	33.2	0.7	0.693	0.017	433.08	99.38
763_26-3_44-47	Nannofossil claystone	32.8	0.3	0.725	0.013	95	4	15.0	0.6	0.700	0.013	421.44	98.13
763B_25-4_111-115	Nannofossil claystone	36.2	0.3	0.737	0.018	164	4	23.6	0.6	0.699	0.018	414.11	97.34
763B_25-1_92-94	Nannofossil claystone	31.7	0.19	0.788	0.011	184	4	30.4	0.6	0.739	0.011	409.42	96.83
763B_24-7_6-9	Clayey Nannofossil chalk	32.51	0.17	0.805	0.010	223	4	36.0	0.6	0.747	0.010	407.71	96.65
763B_24-4_134-137	Clayey Nannofossil chalk	45.7	0.3	0.852	0.011	676	16	78	2	0.727	0.012	404.84	96.34
763B_24-1_56-58	Clayey Nannofossil chalk	33.95	0.11	0.785	0.008	115	2	17.7	0.3	0.757	0.008	399.56	95.77
763_23-4_76-78	Clayey Nannofossil chalk	39.3	0.2	0.716	0.008	104	5	13.8	0.6	0.694	0.008	394.76	95.25
763B_23-1_24-27	Clayey Nannofossil chalk	24.88	0.13	0.762	0.009	38	2	8.0	0.4	0.749	0.009	389.74	94.71
763B_22-1_104-106	Clayey Nannofossil chalk	34.4	0.2	0.542	0.011	104	2	15.4	0.4	0.518	0.011	381.04	93.43
